# Consumption of Non-Prescribed Drugs in Portugal During the Pandemic in 2021

**DOI:** 10.3389/ijph.2023.1606021

**Published:** 2023-07-20

**Authors:** Aida Isabel Tavares, Pedro Lopes Ferreira, Vitor Raposo, Carlota Quintal

**Affiliations:** ^1^ ISEG, UL - Lisbon School of Economics and Management, University of Lisbon, Lisbon, Portugal; ^2^ CEISUC - Centre for Health Studies and Research, University of Coimbra, Coimbra, Portugal; ^3^ FEUC - Faculty of Economics, University of Coimbra, Coimbra, Portugal; ^4^ CEBER - Centre for Business and Economics Research, University of Coimbra, Coimbra, Portugal

**Keywords:** behind-the-counter drugs, nonprescription drugs, social determinants, Portugal, COVID-19

## Abstract

**Objectives:** Portugal liberalised the over-the-counter drugs market in 2005 and provides universal healthcare coverage in a mainly Beveridge-type health system. However, the COVID-19 pandemic has forced healthcare to change how services were delivered, especially increasing remote consultations in primary care. This analysis aims to find the drivers for taking non-prescribed drugs during the pandemic in Portugal. Specifically, it seeks to understand the role of taking prescribed drugs and attending remote medical appointments in the self-medication decision.

**Methods:** In this observational study, we used data collected during the pandemic in Centre Region of Portugal and estimated logistic regression for the whole sample and stratified by sex.

**Results:** The main findings show that people taking prescribed medications and attending a remote consultation are more likely to take non-prescribed drugs. Also, reporting unmet healthcare needs seems to motivate people to choose self-medication.

**Conclusion:** Policy implications are pointed out concerning the health risks raised from self-medication, the role of the pharmacist advising non-prescribed drugs, and the related health risks arising from unmet healthcare needs.

## Introduction

Portugal’s non-prescribed or over-the-counter (OTC) drugs market was liberalised in 2005. Since then, these drugs can be sold in community pharmacies, retailers, supermarkets and other drug outlets [1]. Accompanying this change, promoting self-care and self-medication [2], an individual decision to treat self-recognised illnesses or symptoms without consulting a doctor has naturally contributed to increasing the OTC market. The advantages of this expansion are several: saves the cost of time doctors spend taking care of minor illnesses, such as pain, cold and flu, and allergies [3], saves people time to attend medical attention and expenditure, empowers people over their health and benefits their labour productivity. However, some disadvantages include wrong diagnosis, polypharmacy, adverse reactions and drug interactions, overdose and delay of the correct diagnosis by masking other severe health conditions [2].

The OTC drugs market in Portugal currently represents about a quarter of the pharmaceutical market in volume. About 23% of OTC are sold in retailers, supermarkets and drug outlets; the remaining share is in community pharmacies [4].

The liberalisation of the OTC market also needs to be framed in the Portuguese health system. It is described as a Beveridge type of national health service (NHS) with universal coverage, but it also includes some Bismarkian features [5, 6]. NHS faces several challenges and difficulties, such as waiting lists, unreasonable time to get a medical appointment, unavailability of general practitioners, unmet health needs, and high cost-sharing of prescribed pharmaceuticals, some of which have been exacerbated with COVID-19 in 2020–21.

A recent study [7] of data collected in Portugal before the pandemic in 2019 showed that unmet healthcare needs, due to financial constraints and long distance to health units, were motivating people to substitute healthcare for OTC drugs, while unmet healthcare due to waiting lists had the opposite effect, demotivating people to take those drugs. Other factors such as being female, young, more educated, living in urban areas, suffering from chronic pain, or sleeping difficulties were associated with taking OTC.

During the pandemic 2020–21, several mitigation policies were implemented in Portugal, such as lockdown measures [8]. As a result, face-to-face appointments in primary care decreased significantly (about 38%) in 2020. However, they recovered slightly in 2021 (about 14%), while the remote appointments registered a substantial increase (about 100% in 2020 and 8% in 2021) [9]. So, the pandemic experience brought not only the difficulty in accessing medical appointments in primary care but also a new way of accessing and receiving healthcare through remote appointments. On the other hand, community pharmacies never closed down during the pandemic; they had an essential role in this period, testing and providing hospital drugs to in-home patients. So, the pharmacist was often the closest healthcare professional to whom people could access [10].

This work has a two-fold aim. Firstly, to update evidence concerning the drivers for taking non-prescribed drugs during one peak of the pandemic (November 2021–February 2022), and secondly, to understand what new factors may be associated with taking non-prescribed drugs in Portugal, specifically the role of taking prescribed drugs and attending remote medical appointments.

For this purpose, we resort to the Andersen’s Behavioral Model of Health Services Use, which describes the factors that may influence the use of health services according to individual and contextual levels and three categories of factors, namely, predisposing, enabling and need factors. This conceptual framework has been used to support different empirical studies, including those focusing on buying and taking OTC drugs and self-medication [11–17].

**FIGURE 1:**
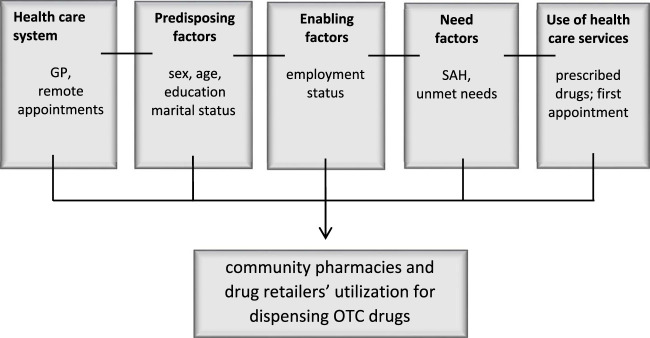
Adapted conceptual framework of Andersen’s Behavioural Model of Health Services Use (Portugal, 2023).

Figure 1 shows the adapted framework of Andersen’s Model to our study and the different available indicators used to express the factors influencing people’s decision to access community pharmacies or other drug retailers for obtaining OTC drugs [18].

## Methods

### Data and Sample

The sample was obtained from a questionnaire implemented between 02 November 2021 and 18 February 2022 in users from primary healthcare units during one of the pandemic peaks (see Supplementary Graph SM1). These 147 health units are located in the Centre Region of Portugal. The response rate was 99.7%, with 7,126 valid answers. The questionnaire aimed to monitor people’s satisfaction towards the services provided by primary healthcare units. It is based on the measurement instrument EuroPeP [19, 20] and validated for Portugal.

Additionally, some other questions were asked to gather information for scientific purposes. For instance, it was asked about unmet healthcare needs and taking drugs following the format used in other international surveys, such as the European Health Survey and the Survey of Health Ageing and Retirement in Europe (SHARE). The Regional Health Authority Directive Board and Ethics Committee approved questionnaire’s implementation.

#### Outcome Variable

The outcome variable for this analysis is labelled OTC, meaning consumption of non-prescribed drugs. This variable is obtained from the question “in the last 2 weeks, have you taken or used any drug, natural product or vitamins which were not prescribed by a doctor?”. OTC equals 1 if the respondent answered yes; it is 0 otherwise.

#### Independent Variables

The set of independent variables used to explain taking OTC is described in Table 1. These variables are grouped according to the categories presented by the Andersen’s model (Figure 1): predisposing factors, enabling factors, need factors (including the unmet needs), use of healthcare services (focusing on the utilization of medical appointments and prescribed drugs), and the healthcare system (capturing organization of services concerning general practictioner accessibility and availability of remote appointments). These two last groups of variables interact with the remaining factors, influencing people’s decision to look for OTC drugs.

**TABLE 1:** Description of independent variables (Portugal, 2023).

Variables name	Description
Predisposing factors	
Age	Number of years old
Female	Dummy variable: 1 if female; 0 if male
Education	The number of years of completed school
Single	Dummy variable: 1 if single; 0 otherwise
Married	Dummy variable: 1 if married; 0 otherwise
Divorced	Dummy variable; 1 if divorced; 0 otherwise
Widow	Reference category for family status
Enabling factors	
Employed	Dummy variable; 1 if employed; 0 otherwise
Unemployed	Dummy variable; 1 if unemployed; 0 otherwise
Student	Dummy variable; 1 if student; 0 otherwise
Other	References category for employment status, including sickness or disability leave, domestic work, volunteer work
Needs Factors	
SAH	Self-assessed health. Ordinal variable ranging from 1 to 5, where 1 means very good and 5 means very bad
Unmet healthcare needs	
unmet_unable_to_get	Dummy variable; 1 if respondent needed a medical appointment but was unable to get it; 0 otherwise. Based on the question “In the last 12 months, did you need a medical consultation but were not able to get an appointment?”
unmet_gave_up	Dummy variable; 1 if respondent gave up a scheduled medical appointment; 0 otherwise. Based on the quesiton “In the last 12 months, did you give up a scheduled appointment?”
unmet_re-schedule	Dummy variable; 1 if respondent had its appointment rescheduled to another date; 0 otherwise. Based on the quesiton “In the last 12 months, did you have a scheduled appointment which was delayed and rescheduled to another date?”
Use of healthcare services	
prescribed_drugs	Dummy variable; 1 if respondent has taken prescribed drugs in the previous 2 weeks; 0 otherwise. Based on the quesiton “In the last 2 weeks, did you take any prescribed drug?”
first_appoint	Dummny variable; 1 if respondent is attending a first consultation in the healthcare unit; 0 otherwise. Based in the question “Is this your first appointment in this healthcare unit?”
Health care system	
GP_assigned	Dummy variable; 1 if respondent is assigned or registered with a General Practitioner (GP); 0 if respondent has no assigned GP. Based on the quesiton “Are you registered with a GP?”
remote_appoint	Dummy variable; 1 if respondent benefited from remote appointment with a healthcare professional from the respecitive healthcare unit; 0 otherwise. Based on the question “In the last 12 months, did you have a remote appointment with a health professional from this healthcare unit?” Further details in Supplementary Note S1

### Statistical Analysis

We started to perform a descriptive analysis of the data. Then we estimated a logistic regression for all the sample and separately for women and men because empirical evidence supports sex differences concerning healthcare use [21–23]. We also performed i) the *VIF test* to check multicollinearity across independent variables, ii) the *linktest* for testing specification errors, and iii) the *Hosmer-Lemeshow test* for model goodness-of-fit. Results are reported using odds-ratio (OR), 95% confidence intervals (CI) and the correspondent *p-values*. We computed predictive probabilities and margin effects for the results concerning the new effects in this analysis, specifically those resulting from taking prescribed drugs and attending a remote medical appointment.

All the analyses presented in this work were performed in STATA 15.

## Results

Regarding the consumption of OTC drugs, there were 1,427 positive responses; that is, about a fifth of the sample (20.6%) had taken those drugs in the two previous weeks to the questionnaire implementation. Before the pandemic [7], that percentage was about 22.5%.

Descriptive statistics for independent variables are displayed in Table 2. The majority of the sample were women and the average age was about 50 years old; the majority was married, had an average of 7 years of education and were employed; about 40% of people reported a good or very good health status, but also nearly 40% reported taking prescribed drugs and had benefited from a remote consultation; finally, about 30% of people reported some unmet healthcare needs. In the Supplementary Table SA1, some of these descriptive statistics are compared with those presented for the study before the pandemic [7].

**TABLE 2:** Descriptive statistics for independent variables (Portugal, 2023).

Variables name	Descriptive statistics
Predisposing factors	
Age (Mean, Standard Deviation)	50.9 (16.5)
Female (Number, %)	4,550 (64.2%)
Education (Mean Years, Standard Deviation)	7 (4,2)
Single (Number, %)	1,277 (18.0%)
Married (Number, %)	4,676 (65.9%)
Divorced (Number, %)	494 (7.0%)
Widow (Number, %)	646 (9.1%)
Enabling factors	
Employed (Number, %)	4,405 (62.3%)
Unemployed (Number, %)	481 (6.8%)
Student (Number, %)	228 (3.2%)
Needs Factors	
SAH (level, number, %)	[1] very good 577 (8.1%) [2] good 2,820 (39.8%) [3] reasonable 3,202 (54.1%) [4] bad 441 (6.2%) [5] very bad 53 (0.8%)
Unmet healthcare needs	
unmet_unable_to_get	2,083 (30.3%)
unmet_gave_up	559 (8.0%)
unmet_re-schedule	1,998 (29.1%)
Use of healthcare services	
prescribed_drugs	2,757 (39.4%)
first_appoint	679 (9.5%)
Healthcare system	
GP_assigned	6,688 (93.9%)
remote_appoint	2,704 (38.0%)

The results from the estimated logistic regression are presented in Table 3, for all sample and stratified by sex. In addition, the specification test *(linktest*) and the goodness-of-fit test (*Hosmer-Lemeshow*) show that the model is well-specified and has a high goodness-of-fit. Finally, the *VIF test* indicates the absence of multicollinearity.

**TABLE 3:** Estimated logistic regressions (Portugal, 2023).

	All sample				Male sample				Female sample			
	OR	95% CI		P>z	OR	95% CI		P>z	OR	95% CI		P>z
Predisposing factors												
Age	0.998	0.991	1.004	0.448	0.999	0.987	1.010	0.797	0.998	0.991	1.005	0.615
Female	**1.246**	**1.089**	**1.426**	**0.001**								
Education	**1.068**	**1.047**	**1.090**	**<0.001**	**1.062**	**1.024**	**1.102**	**0.001**	**1.070**	**1.045**	**1.096**	**<0.001**
Single	0.878	0.629	1.225	0.444	**0.475**	**0.269**	**0.840**	**0.010**	1.155	0.766	1.741	0.492
Married	0.829	0.633	1.084	0.170	**0.403**	**0.259**	**0.627**	**<0.001**	1.137	0.813	1.589	0.453
Divorced	1.040	0.959	1.128	0.347	**0.837**	**0.723**	**0.969**	**0.017**	**1.137**	**1.030**	**1.255**	**0.011**
Enabling Factors												
Employed	**0.771**	**0.630**	**0.944**	**0.012**	0.800	0.560	1.144	0.222	**0.770**	**0.600**	**0.986**	**0.038**
Unemployed	**0.694**	**0.507**	**0.950**	**0.023**	0.937	0.506	1.729	0.836	**0.639**	**0.442**	**0.924**	**0.017**
Student	1.084	0.710	1.654	0.709	0.962	0.439	2.108	0.923	1.181	0.711	1.965	0.521
Needs												
SAH	1.005	0.917	1.104	0.898	0.975	0.817	1.162	0.773	1.023	0.917	1.142	0.681
Unmet healthcare needs												
unmet_unable_to_get	**1.447**	**1.261**	**1.660**	**<0.001**	**1.243**	**0.970**	**1.592**	**0.086**	**1.550**	**1.311**	**1.831**	**<0.001**
unmet_gave_up	1.216	0.980	1.510	0.076	1.005	0.666	1.516	0.983	1.280	0.988	1.658	0.062
unmet_re-schedule	**1.193**	**1.036**	**1.374**	**0.014**	1.037	0.811	1.326	0.772	**1.274**	**1.071**	**1.516**	**0.006**
Use of healthcare services												
prescribed_drugs	**1.589**	**1.386**	**1.822**	**<0.001**	**1.747**	**1.356**	**2.252**	**<0.001**	**1.553**	**1.319**	**1.827**	**<0.001**
first_appoint	0.959	0.769	1.194	0.706	1.051	0.734	1.505	0.785	0.909	0.688	1.201	0.504
Healthcare system												
GP_assigned	0.960	0.739	1.248	0.762	0.818	0.531	1.260	0.363	1.049	0.756	1.457	0.774
remote_appoint	**1.229**	**1.083**	**1.393**	**0.001**	**1.282**	**1.026**	**1.601**	**0.029**	**1.213**	**1.041**	**1.415**	**0.013**
_cons	0.094	0.049	0.178	<0.001	0.181	0.059	0.557	0.003	0.082	0.039	0.174	<0.001
Number of obs	6,489				2,321				4,168			
Wald chi2	187.810				54.580				147.390			
Prob > chi2	<0.001				<0.001				<0.001			
Pseudo R2	0.030				0.026				0.033			
Log pseudolikelihood	−3,189.77				−1,049.166				−2,127.53			
Linktest											
hatsq (P>z)	0.059	(0.637)			0.264	(0.214)			−0.188	(0.187)		
Hosmer-Lemeshow												
chi2 (Prob > chi2)	3.850	(0.870)			5.490	(0.705)			6.920	(0.546)		
VIF test (mean)	1.84											

Note: in bold statistically significant results.

In summary, firstly, concerning predisposing factors for taking OTC, we found that women and more educated people are more likely to take OTC than men and than less educated ones. However, we also found some differences between sexes. For example, while marital status may be relevant for men to choose OTC, the same does not happen with women, except for the case of divorced, where the odds-ratio is larger than one.

Secondly, concerning enabling factors, we found a difference across sexes: while professional status is significant for women, it is not for men (no statistically significant odds ratio). Also employed or unemployed women are less interested in taking OTC than individuals with other employment status.

Thirdly, self-assessed health, used as a proxy for the need for OTC drugs, is not significant in any model estimation.

Fourthly, unmet healthcare needs do matter for taking OTC. People reporting unsatisfied needs also report a higher likelihood of taking OTC (OR > 1). This situation is, however, more significant for women than men, for whom only the inability to get an appointment is statistically significant.

Finally, two interesting and statiscially significant results: i) individuals taking prescribed drugs are more likely to take OTC drugs (OR = 1.589 for all sample), as well as ii) individuals having a remote consultation (OR = 1.229 for all sample).

In this last part of the results section, we present the predictive probabilities and marginal effects for the probability of taking OTC dependent on what happens to the independent variables, specifically, taking prescribed drugs and benefiting from a remote consultation. A summary of these marginal effects is presented in Table 4.

**TABLE 4:** Marginal effects (Portugal, 2023).

	At means	prescribed_drugs = 1	remote_appoint = 1
Prob (OTC = 1) (*p-value*)	0.196 (<0.001)	0.226 (<0.001)	0.216 (<0.001)
Δ Prob (OTC = 1) (*p-value*)		0.07 (<0.001)	0.033 (0.002)
N = 6,489			

Note: “at means” translates the mean value of all the independent variables to make the calculus; and “Δ” translates the increase in the probability.

### Looking at This Table

i. The probability that a person takes OTC is about 19.6% (*p*-value < 0.001), given that all the independent variables are set at their mean value.
ii. The probability that a person takes OTC is equal to 22.6% (*p*-value < 0.001), given that s/he takes prescribed drugs with all the remaining independent variables set at mean value.
iii. The probability that a person takes OTC is equal to 21.6% (*p*-value < 0.001), given that s/he attended a remote appointment with all the remaining independent variables set at a mean value.
iv. The increase in the probability that s/he takes OTC is equal to 7% (*p*-value < 0.001), given that s/he takes prescribed drugs with all the remaining independent variables set at a mean value.
v. The increase in the probability that s/he takes OTC is equal to 3.3% (*p*-value = 0.002), given that s/he attended a remote appointment with all the remaining independent variables set at a mean value.

So, the marginal effect of taking prescribed drugs (iv) is larger than that of attending a remote appointment (v).

## Discussion

After liberalising the OTC drugs market in Portugal, taking OTC became more widespread, with well-documented advantages and disadvantages. However, empirical evidence for the factors associated with taking OTC in European countries is short. A recent study for Portugal, before the pandemic, in 2019, suggested some drivers for taking non-prescribed drugs for self-care. The analysis here presented aims to update the results after the pandemic and advance additional drivers for taking prescribed medications and attending remote medical appointments.

### Key Findings

The key findings of this work point, firstly, people already taking prescribed medicines or attending remote medical appointments are more likely to take non-prescribed drugs. Secondly, unmet healthcare needs seem to be a driver for taking non-prescribed drugs. Finally, when stratifying the sample by sex, some statistically significant results for women are not equally found for men.

### Interpretation

This work’s first major significant finding is that people who attended a remote medical appointment or were taking prescribed drugs presented a higher likelihood of taking non-prescribed drugs. And the increased probability of taking an OTC drug is slightly higher for people already taking prescribed medications than for those who attended a remote appointment. These findings are new evidence of how people behave under a constraint situation of a pandemic.

It is worth, though, analysing if this behaviour continues or will change in the future. In the public health crisis scenario of COVID-19, people were being constrained to stay at home or to work under strict conditions. Without the human contact of health professionals and given prescribed drugs needed (and prescriptions were often obtained from an online or digital prescription), the pharmacist was the closest human contact people could afford, which could motivate people to take OTC drugs [10, 24].

The second major finding concerns to the role of unmet healthcare needs as a driver for taking OTC drugs; this result was also found before the pandemic in Portugal [7]. The results previously found showed that people were more likely to take non-prescribed drugs if they could not access healthcare for financial constraints and excess distance. However, people on waiting lists were less likely to choose OTC.

The evidence obtained in this research presents a slightly different pattern. People who need healthcare but cannot get an appointment, being on a waiting list, and people who have an appointment postponed, forced to be on hold, are motivated to take OTC. So, during the pandemic, when healthcare units struggled to deal with the public health crisis and the access to healthcare was difficult, people seemed to be substituting doctors for pharmacists and choosing to take non-prescribed drugs. Although this is a reasonable choice during the pandemic, the learning process may result in a permanent substitution between doctors and pharmacists. This scenario calls for future research to understand the dynamics of people choosing to take OTC drugs. On the other hand, another concern for this situation is that the absence of healthcare and the masking of symptoms with these drugs may delay a diagnosis or worsens health conditions, which could be prevented. This is one of the major problems raised with the pandemic and the incapacity of response of the health system to attend to all needs at that time.

Thirdly, as found in previous empirical studies [25–27], women or people with higher education are more likely to take OTC medicines. Despite the absence of information concerning people’s income, the level of education may be a good proxy for the available income so that higher education can be associated with higher income. People with higher income are also more likely to buy non-prescribed drugs. People with higher education levels are more endowed with personal resources (cognitive, informative and relational) that may empower people in their health [28–32].

On the other hand, people who are either employed or unemployed are less likely to take OTC drugs. Given the pandemic context and lockdown measures, it is reasonable to expect that people, no matter their labour status, may adopt identical behaviours concerning self-medication. However, the reasons supporting these behaviours may be different. For example, while employed people, who work at home or in essential services, fear to mask COVID-19 illness; unemployed people may be financially constrained to afford to buy non-prescribed drugs.

Finally, we refer to the sex differences found in our estimations [33]. Men are more influenced by predisposing factors. While education positively contributes to OTC drug consumption, single, divorced or widowed men are less likely to buy those medicines compared to married men. Women seem less associated with these factors, while education and divorce are the only motivating factors to take OTC. Conversely, to men, women are more prone to be influenced by enabling factors. While employed and unemployed women take OTC; the same association cannot be found for men. One possible explanation for this evidence comes from the learning experience related to accessing and using pharmacy services that women have from buying contraceptive pills [7, 34].

### Strengths and Limitations

The major strength of this work is the additional contribution given to previous research exploring the drivers of OTC drug buying in European countries.

Some limitations, though, are to be addressed. First, the questionnaire asks if people took drugs that a physician did not prescribe. This question may have a biased interpretation. People may consider taking medications from the bathroom cabinet that were prescribed long before or medicines prescribed for a family member or a friend as possible answers.

Second, the questionnaire question focuses on taking OTC drugs 2 weeks before. Given that questionnaires were applied during the winter, it could be that people were more likely to take flu-related OTC drugs. However, if it were implemented during the spring or summer, people would be taking allergy-related drugs. In this way, we do not expect a strong seasonal bias in collected data.

Third, data were collected inside primary healthcare units in the Centre Region of Portugal. It may be argued that this sample may not represent the Portuguese population. Nevertheless, the main differences between those two geographic spaces are in the ageing index and the unemployment rate. While Portugal has an ageing index of 184.6, in Centre Region it is equal to 228.6, and the unemployment rate is slightly lower in Centre Region (6.6% in Portugal and 5.8% in Centre Region) [35]. However, age was not found to be a driver of OTC, and both labour conditions of employment and unemployment were found to be statistically significant, so the results found for Centre Region may not be so different from those that would be found for Portugal. In the Supplementary Table SM1, we present the different descriptive statistics across the previous study [7] and Census 2021 for Centre Region and Portugal.

Finally, the analysis performed in this work cannot be interpreted as causal relationships. The analysis provides correlations and predicted probabilities related to taking OTC by people. For a causal analysis, different data and methods would be needed.

### Policy Implications

The policy implications that arise from our results are mainly two-fold. Firstly, the concern for the substitution that people seem to be making between the absence of access to healthcare and taking non-prescribed drugs for self-medicating. This substitutive decision raises health risks resulting from wrong decisions on self-care for what seems a minor illness.

Secondly, the complementarity between remote appointments, specially those for requesting prescriptions or having a medical consultation and taking OTC drugs. While a certain value is added from this sort of non-presential appointment, there is an issue concerning human relationships. Further research needs to be followed to understand to what extent the absence of direct or close human contact may contribute to increased health empowerment perception and overconfidence by people, which may not be advantageous for a timely correct diagnosis.

### Conclusion

Introducing remote medical appointments seems to be a new driver for people taking non-prescribed drugs. Further studies need to be conducted to monitor this behaviour and understand the pharmacist’s role in this choice. Additionally, the prevalence of unmet healthcare needs may increase the likelihood of people choosing OTC drugs, which may not be cost-effective from a societal and individual perspective. Therefore, one future policy measure that significantly impacts OTC drugs is the reduction of unmet healthcare.
