# Supplementary material for: Consumption of Non-Prescribed Drugs in Portugal During the Pandemic in 2021
Source: Int J Public Health. 2023 Jul 20;68:1606021. doi: 10.3389/ijph.2023.1606021 (PMC10397401; doi:10.3389/ijph.2023.1606021)
Supplement: Supplementary file 1 [file DataSheet2.docx]

**Appendix**

“Graph A1: Daily new confirmed COVID-19 cases in Portugal (Portugal, 2023)”.


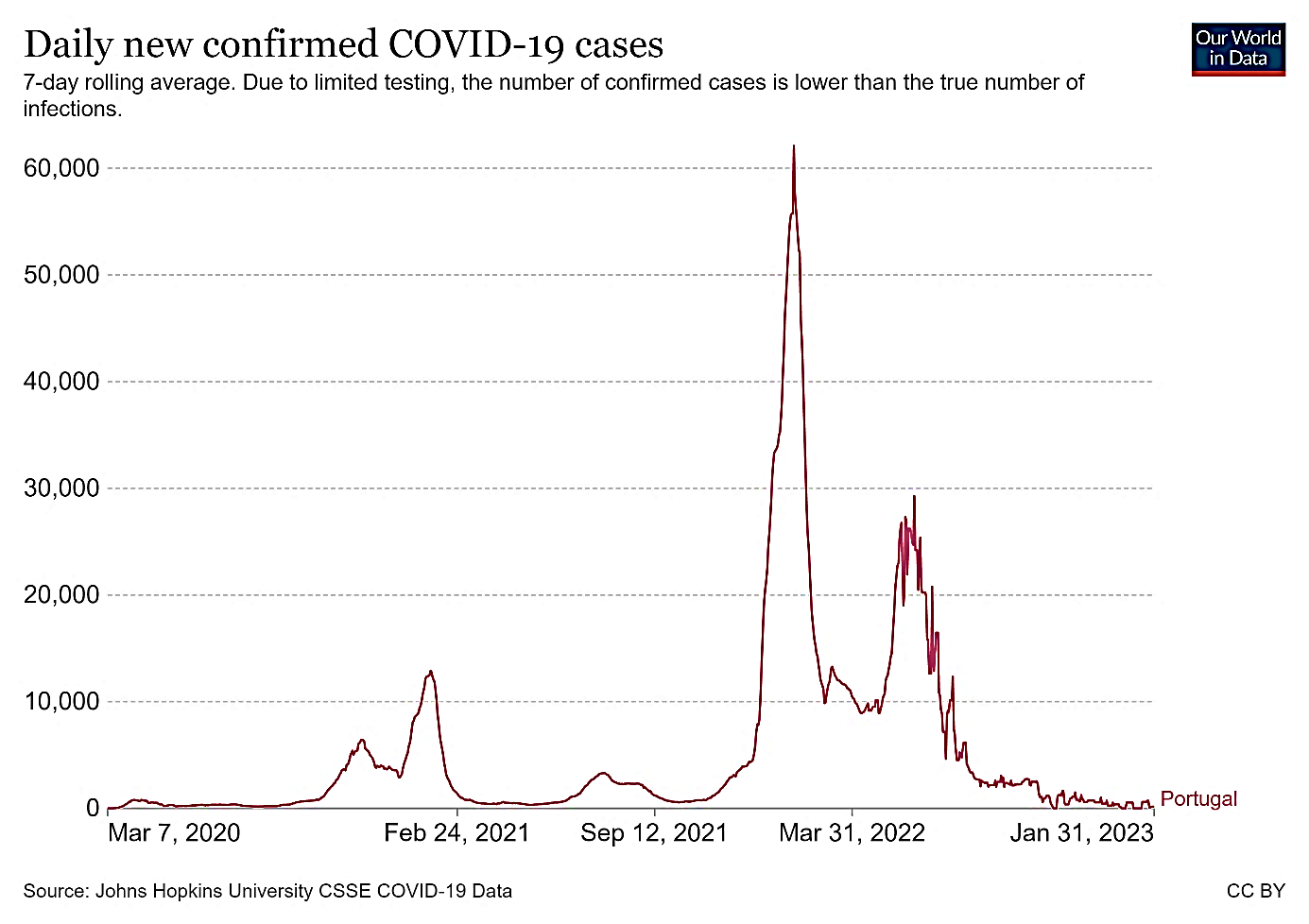


Source: Mathieu E, Ritchie H, Rodés-Guirao L, et al. (2020) Coronavirus Pandemic (COVID-19). Published online at OurWorldInData.org (2022).

Reference: Mathieu E, Ritchie H, Rodés-Guirao L, et al. Coronavirus Pandemic (COVID-19); 2020. Published online at OurWorldInData.org:2022. Available at https://ourworldindata.org/coronavirus/country/portugal. Accessed 5^th^ January 2023.

“Table A1: Comparative descriptive statistics (Portugal, 2023)”.

| **Variables name** | **Descriptive statistics** | **Tavares et al. 2022** | **CENSUS 2021**  **NUTS II Centre** | **CENSUS 2021 Portugal** |
| --- | --- | --- | --- | --- |
| **Demographic** | % | % | % | % |
| age +65 | 20.0 | 37.5 | 27.0 | 23.4 |
| female | 64.2 | 65.6 | 52.4 | 52.4 |
| schooling 9 years | 44.4 | 25.8 | 52.5 | 49.7 |
| single | 18.0 | 23.9 | 39.5 | 43.5 |
| married | 65.9 | 50.1 | 44.1 | 41.0 |
| divorced | 7.0 | 10.1 | 7.7 | 8.0 |
| widow | 9.1 | 15.6 | 8.7 | 7.5 |
| **Labour status** |  |  |  |  |
| employed | 62.3 | 45.0 | na | na |
| unemployed | 6.8 | 7.0 | 5.8 | 6.6 |
| student | 3.2 | 3.0 | na | na |
| **SAH** |  |  |  |  |
| [1] very good  [2] good  [3] reasonable  [4] bad  [5] very bad | 8.1  39.8  54.1  6.2  0.8 | 9.8  33.3  40.9  12.2  3.8 | na | na |

na: not-available
