# Supplementary material for: Consumption of Non-Prescribed Drugs in Portugal During the Pandemic in 2021
Source: Int J Public Health. 2023 Jul 20;68:1606021. doi: 10.3389/ijph.2023.1606021 (PMC10397401; doi:10.3389/ijph.2023.1606021)
Supplement: Supplementary file 2 [file DataSheet1.docx]

**Supplementary Material**

Supplementary Material Note 1

One remark concerning variable "remote_appoint". The survey question for this variable is "in the last 12 months, did you have a non-presential appointment with a health professional of this healthcare unit?". The remote appointment question includes the following non-exclusive cases: requestion drug prescriptions (62%), requesting diagnostic tests (53%), showing diagnostic tests (50%), medical consultation (68%), nurse consultation (35%), questions and doubts on COVID-19, or other non-specified situations (not assessed in the survey).
